# Optimizing the Interventional Approach for Embolization of Pulmonary Arteriovenous Malformations in Patients With Osler-Weber-Rendu Syndrome

**DOI:** 10.7759/cureus.9878

**Published:** 2020-08-19

**Authors:** Joseph R McFarland, Taylor S Harmon, Tiffiny Hunsaker, Gunvir Gill, Arya N Bagherpour

**Affiliations:** 1 Radiology, University of Texas Medical Branch, Galveston, USA; 2 Interventional Radiology, University of Florida College of Medicine, Jacksonville, USA; 3 Interventional Radiology, University of Texas Medical Branch, Galveston, USA

**Keywords:** pulmonary artery, arteriovenous malformations, osler-weber-rendu, hereditary hemorrhagic telangiectasia, embolization, interventional radiology, amplatzer plug device, coil embolization, pulmonary angiogram, computed tomography

## Abstract

Techniques in vascular and interventional radiology are adapted to the ever-evolving clinical challenges that interventional operators face. In the case of rare diseases, supporting literature that guides an operator’s plan for intervention is limited. As a result, published case reports and series can be utilized to direct future intervention and potentially help others tasked with similar clinical scenarios. The proceeding case offers an interventional solution to a clinical manifestation of an otherwise rare disease, Osler-Weber-Rendu (OWR) syndrome. The supporting literature for techniques in embolization of pulmonary arteriovenous malformations (AVMs) in OWR syndrome is limited due to disease rarity. Therefore, the objective of the following case is to offer clinical insights on how to perform this procedure successfully and critique methods previously utilized.

## Introduction

Osler-Weber-Rendu (OWR) syndrome, also referred to as Hereditary Hemorrhagic Telangiectasia, is a rare autosomal dominant disease affecting approximately 0.02 percent of the population [1]. Several pathologic manifestations have been reported with OWR syndrome, likely due to the heterogeneity of associated gene mutations and individual environmental exposures encountered [1]. The Curacao criteria describe physical and clinical manifestations that can be utilized to diagnose OWR, including the presence of pulmonary arteriovenous malformations (AVMs) [1].

AVMs are documented in up to 50 percent of diseased individuals [1]. Pulmonary AVMs place patients with OWR at increased risk for complications including hypoxemia, brain abscess, pulmonary hypertension, high-output heart failure, migraine, and hemorrhage [1,2]. Many of these complications are attributed to the tendency of pulmonary AVMs to become physiologic right-to-left shunts, subsequently increasing the susceptibility for paradoxical embolization to occur [3]. The high morbidity and mortality of the associated embolic events has heightened the need for early disease recognition and screening [2]. International guidelines for the management of OWR syndrome have recommended screening patients for AVMs with transthoracic contrast echocardiography, which has higher sensitivity than computed tomography (CT) angiography [4]. Nonetheless, contrast-enhanced chest CT is still widely used for initial screening purposes [3].

Various techniques have been utilized by interventional radiologists for the embolization of vascular pulmonary lesions in patients with rare diseases, such as the embolization of pulmonary artery aneurysms in Behcet’s syndrome [5]. Similar techniques are employed with transcatheter embolization as the treatment of choice for pulmonary AVMs in all adults and symptomatic children with OWR syndrome, as short- and long-term data have proven it to be effective and safe [4]. There are several available embolization device options for this application including coils, vascular plugs, and occlusion balloons [6]. Furthermore, the treatment of pulmonary AVMs reduces migraine headaches, due to the resolution of hypoxemia and decreased passage of vasoactive substances from venous circulation [2].

The following case describes a patient with OWR syndrome who presents with multifocal cerebral abscesses in the setting of several pulmonary AVMs. The case highlights the optimization of standard techniques for embolization of AVMs, and can be used to guide and assist other interventional operators in similar clinical scenarios.

## Case presentation

**Figure 1:**
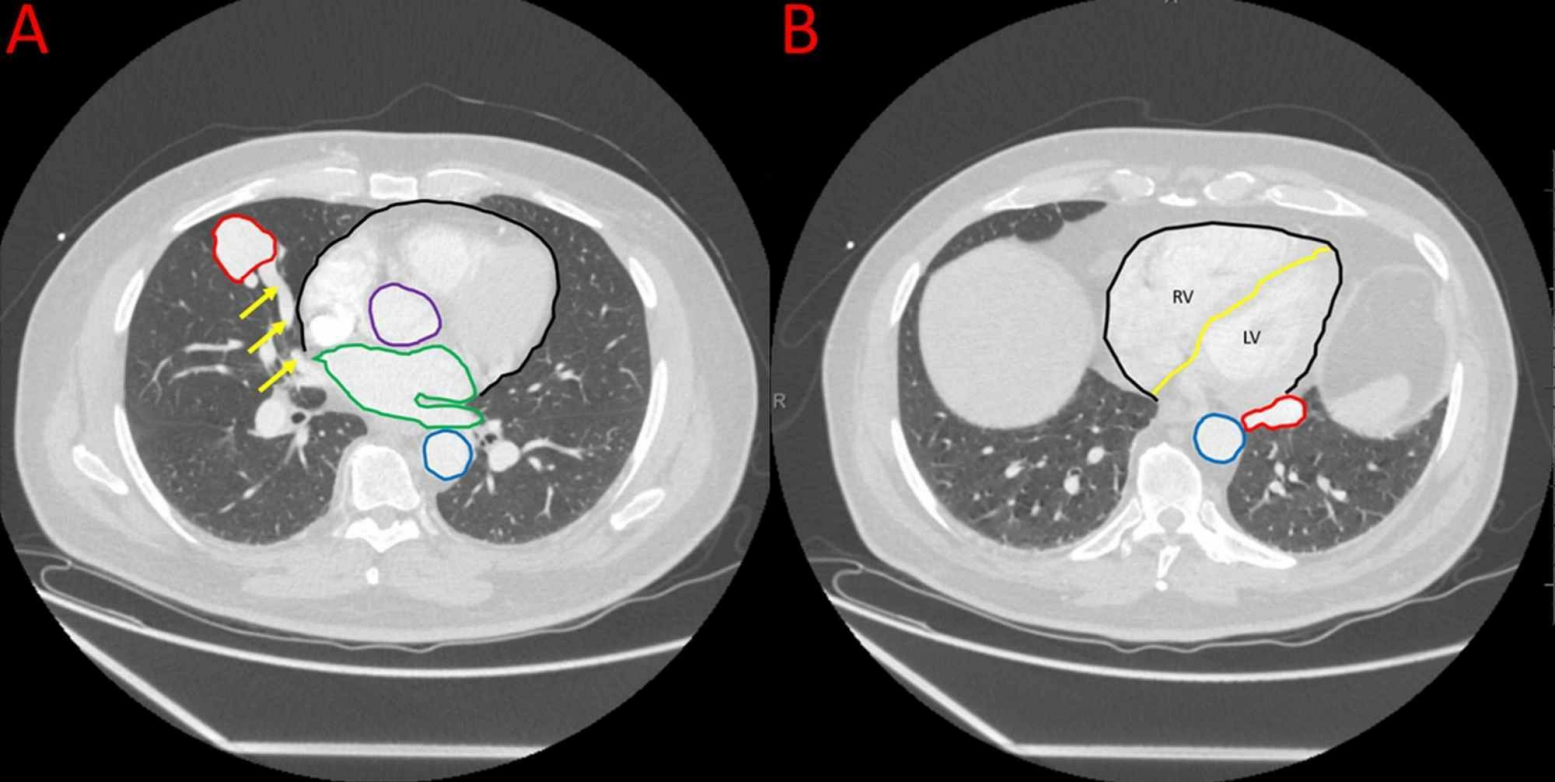
CT of the Chest Showing Two Pulmonary Artery AVMs in the Axial Field of View CT of the chest with contrast in the axial view at different levels, reveals two pulmonary arteries to pulmonary vein fistulas, or AVMs (red circles) with one in the right middle lobe (A) and one in the left lower lobe (B). The aneurysmal component in the right middle lobe (A) measures 2.6 cm × 2.5 cm × 2.8 cm. The AVM is supplied by a single segmental branch of the right pulmonary artery (yellow arrows). The pulmonary veins draining into the left atrium are in the axial field of view, demonstrated by the green outline. The cardiac border can be seen in the axial field, demonstrated by the black outline. The ascending and descending aorta can be seen in the axial field of view, demonstrated by the purple and blue outlines, respectively. The second AVM (B) originates from a single-segmental branch of the pulmonary artery in the left lower lobe and contains a multilobulated aneurysm measuring approximately 1.6 cm × 3.0 cm × 1.7 cm. The cardiac border can be seen in the axial field of view, demonstrated by the black outline. The right and left ventricles of the heart can be seen in the axial field of view, demonstrated by the letters “RV,” and “LV,” respectively. The descending aorta can be seen in the axial field of view, demonstrated by the blue outline. AVM: arteriovenous malformation.

A 60-year-old male with OWR syndrome was hospitalized due to a four-week history of intermittent fever, worsening headaches, and progressive fatigue, in the setting of leukocytosis. Physical examination on admission was remarkable for erythema of his right ankle suggestive of cellulitis, multiple cutaneous telangiectasias, and delayed speech. A chest radiograph showed a 2.9 cm well-circumscribed opacity in the right lower lung, and subsequent CT of the chest with contrast revealing two large pulmonary AVMs (Figure 1).

**Figure 2:**
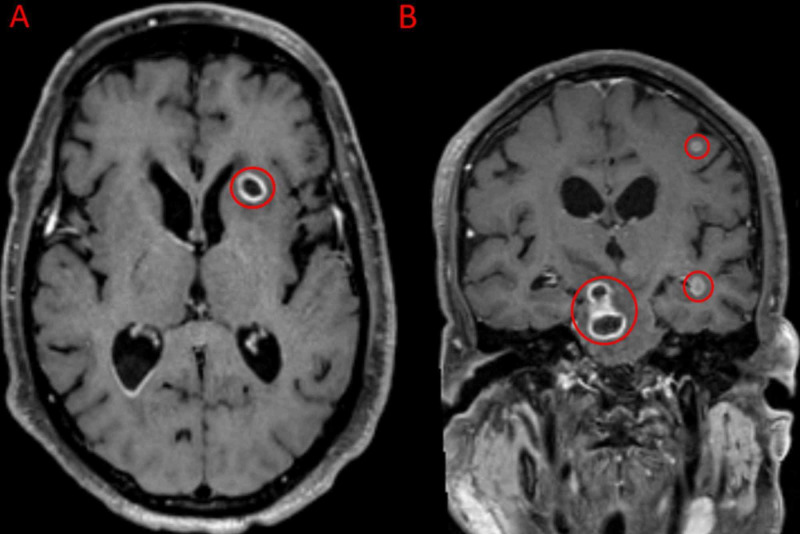
T1 Weighted MRI Demonstrating Concern for Abscesses or Septic Emboli T1 weighted MRI of the brain, post-contrast, in the axial (A) and coronal (B) planes demonstrate multifocal ring-enhancing lesions and hyperintense foci (red circles), concerning for subacute and acute abscesses or septic emboli. The most prominent lesion is located in right hemipons (B, largest red circle) measuring approximately 1.6 cm × 1.5 cm, with mild surrounding hyperintensity likely reflective of vasogenic edema.

Subsequent magnetic resonance imaging (MRI) of the brain was remarkable for possible ventriculitis and multifocal diffusion restriction, concerning for septic emboli or abscesses (Figure 2).

Cerebral spinal fluid and blood cultures were negative, and echocardiography revealed no evidence of endocarditis or a patent foramen ovale. The patient was treated with broad-spectrum antibiotics but continued to worsen clinically. He developed focal left-sided weakness and hyponatremia, in the setting of a right pontine hemorrhage and worsening cerebral edema associated with the intracranial abscesses. Subsequently, the patient was managed in the neurological intensive care unit. After several days of intravenous steroid therapy and electrolyte supplementation, the patient achieved neurological stability and was transferred to the floor. 

Interventional radiology was consulted for the management of pulmonary AVMs, which were attributed to the likely etiology of the intracranial abscesses. Informed consent was obtained and the patient was brought to the interventional suite.

**Figure 3:**
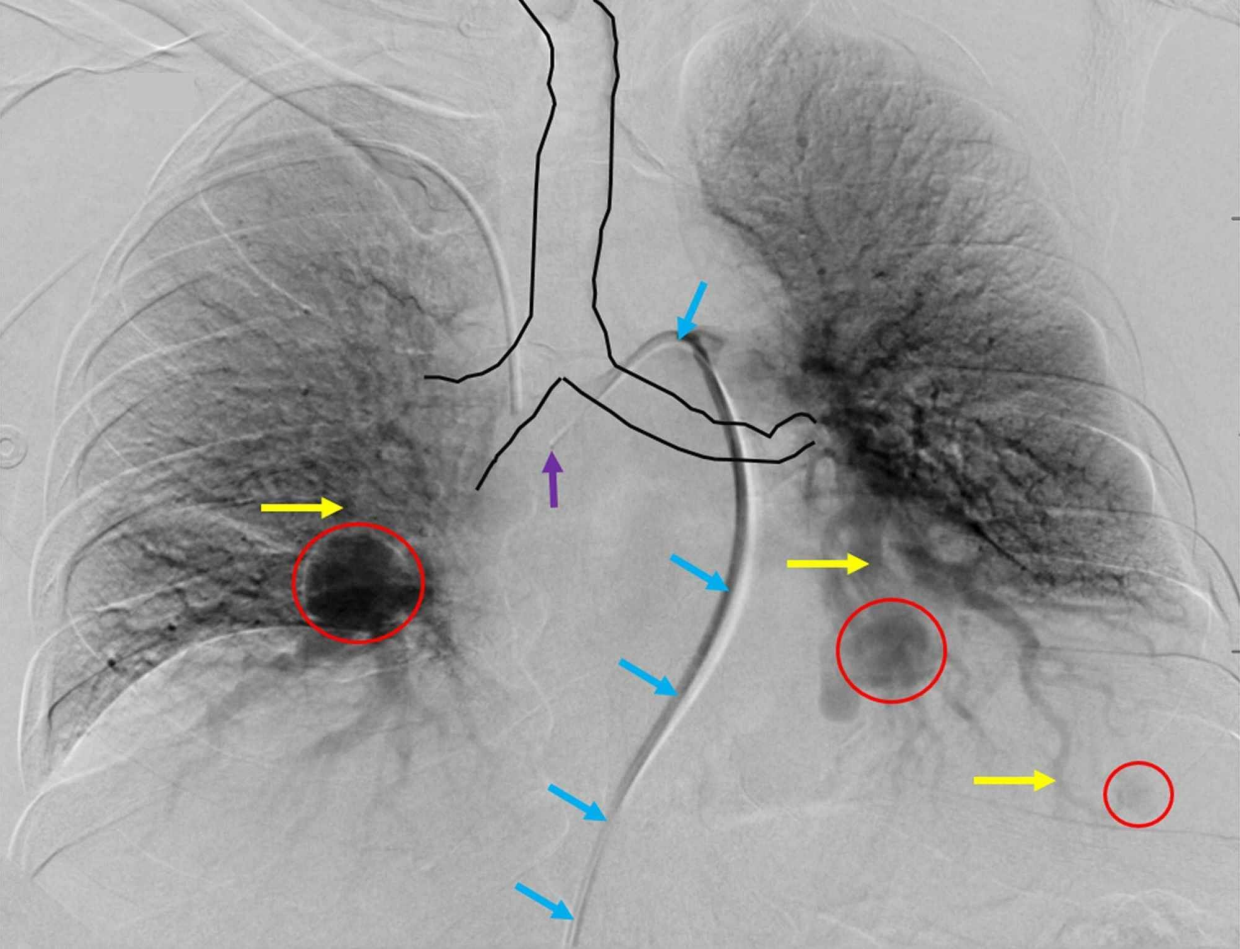
Pulmonary Arteriogram Showing Three Separate AVMs and Respective Supplying Arterial Branches The initial arteriogram from the main pulmonary artery demonstrates three separate AVMs in the medial segment of the right middle lobe, a lateral segment of the left lower lobe, and an anteromedial segment of the left lower lobe (red circles). The two larger AVMs correlated with the observed findings on the initial CT of the chest. An occult AVM (red circle, bottom right of image) was only definitively visualized on CT of the chest, after meticulous retrospective analysis. The yellow arrows demonstrate the pulmonary artery branches that supply the identified AVMs. The blue arrows show the sheath accessing the main pulmonary artery, with the tip of the wire displayed by the purple arrow. The trachea can be seen outlined in black for visual reference.

The right common femoral vein was accessed with a micropuncture needle using ultrasound guidance. After access was made, a 5 French, 10 cm vascular sheath was placed in the right groin. Using fluoroscopic guidance, the main pulmonary artery was selected using a C2TM, 5 French catheter (Boston Scientific, Marlborough, USA) and 0.035-inch GlidewireTM (Terumo Medical, Somerset, USA). The guidewire was exchanged for a 0.035-inch Amplatz Super StiffTM wire (Boston Scientific, Marlborough, USA). The sheath was upsized to a longer 8 French, 70 cm sheath with the placement of the tip of the sheath into the main pulmonary artery. Through the sheath, a diagnostic pulmonary angiogram was performed. An occult third pulmonary artery AVM in the left lower lobe adjacent to the costophrenic sulcus was identified (Figure 3).

After selecting the right middle pulmonary artery using a 0.035-inch Glidewire and 5 French BerensteinTM catheter (Boston Scientific, Marlborough, USA), an arteriogram was performed revealing a large pulmonary AVM within the medial segment of the right middle lobe. The 6.8 mm supplying artery was selected with the Glidewire and Berenstein catheter, after which the catheter was exchanged for a 6 French EnvoyTM catheter (Codman Neuro - Depuy Synthes, Warsaw, USA) over a RosenTM wire (Cook Medical, Bloomington, USA). Embolization was performed using a 12 mm AmplatzerTM vascular plug (St. Jude Medical, Plymouth, USA) which was deployed through the catheter to embolize the supplying artery. A post-embolization angiogram demonstrated successful embolization.

In a similar manner, the left pulmonary artery was selectively catheterized with a subsequent arteriogram demonstrating two AVMs in the left lower lobe; one large AVM was within the anteromedial segment, and the other small AVM was within the lateral basal segment. The 3.8 mm supplying artery for the small AVM was selected with the Glidewire and Berenstein catheter. The catheter was again exchanged over a Rosen wire for a 6 French Envoy catheter. A 6 mm Amplatzer Vascular Plug was deployed into the supplying artery. A subsequent angiogram demonstrated complete embolization.

**Figure 4:**
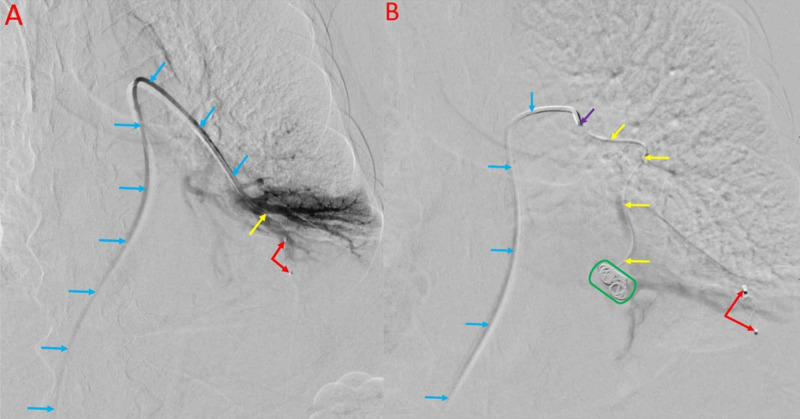
Successful Plug and Microcoil Embolization of Pulmonary AVMs Successful embolization of AVMs within the lateral segment of the left lower lobe using an Amplatzer plug (red arrows) is shown (A). The blue arrows show the access catheter traversing the pulmonary artery, with catheter tip within the lateral segment of the left lower lobe (yellow arrow). Successful embolization of an AVM within the anteromedial segment of the left lower lobe using Terumo detachable microcoils (green circle) is shown (B). The blue arrows show the access catheter traversing the pulmonary artery, with the catheter tip shown (purple arrow). The anteromedial segment of the left lower lobe was accessed with a microcatheter (yellow arrows) and terminates where the detachable microcoils were deployed. Additionally, the Amplatzer plug which embolized the AVM in the lateral segment of the left lower lobe can be seen (red arrows).

The 4.1 mm supplying artery of the anteromedial basal segment of the left lower lobe was selected with a ProgreatTM microcatheter (Terumo Medical, Somerset, USA) and 0.014-inch FathomTM microwire (Boston Scientific, Marlborough, USA). Vessel tortuosity and small catheter requirement prevented the use of an Amplatzer vascular plug for this AVM. Embolization was achieved using three AZUR CXTM detachable coils (Terumo Medical, Somerset, USA) measuring 8 mm, 7 mm, and 5 mm, respectively. A subsequent angiogram demonstrated complete embolization (Figure 4).

**Figure 5:**
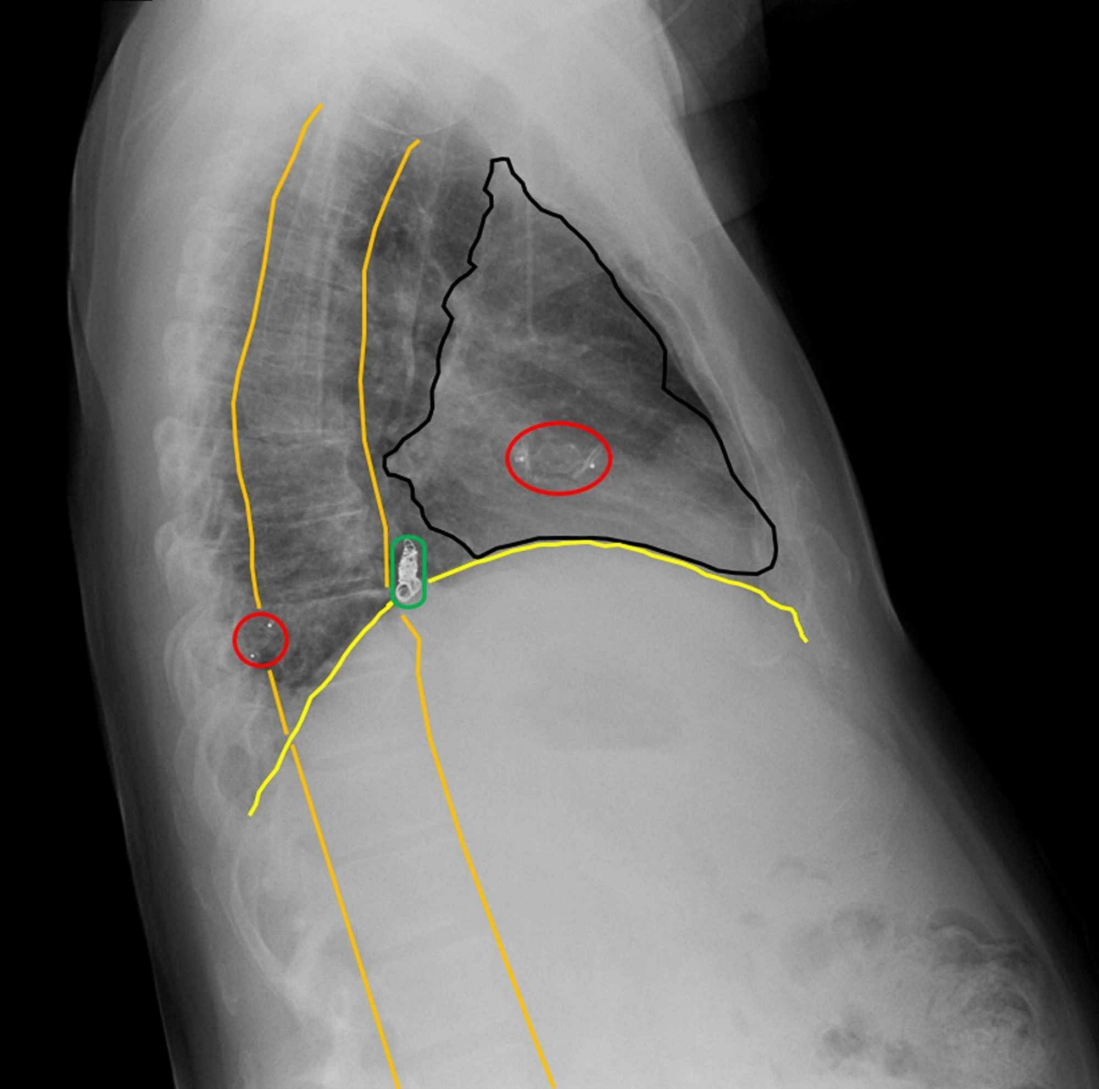
Postoperative Radiograph Showing Appropriately Positioned Embolization Devices Throughout the Chest A postoperative lateral chest radiograph demonstrates Amplatzer plugs (red circles) and Terumo detachable microcoils (green circle) at the locations of the embolized AVMs. The heart border (black outline), diaphragm (yellow line), and vertebral column (orange lines) are demonstrated in the lateral view.

A final post-embolization angiogram from the main pulmonary artery, demonstrated complete embolization of the previously selected feeding arteries, without opacification of the AVMs. All catheters, wires, and sheath were safely removed and hemostasis was achieved. The patient tolerated the procedure well without any complications. A postoperative radiograph demonstrated coils and vascular plugs in the expected position (Figure 5).

**Figure 6:**
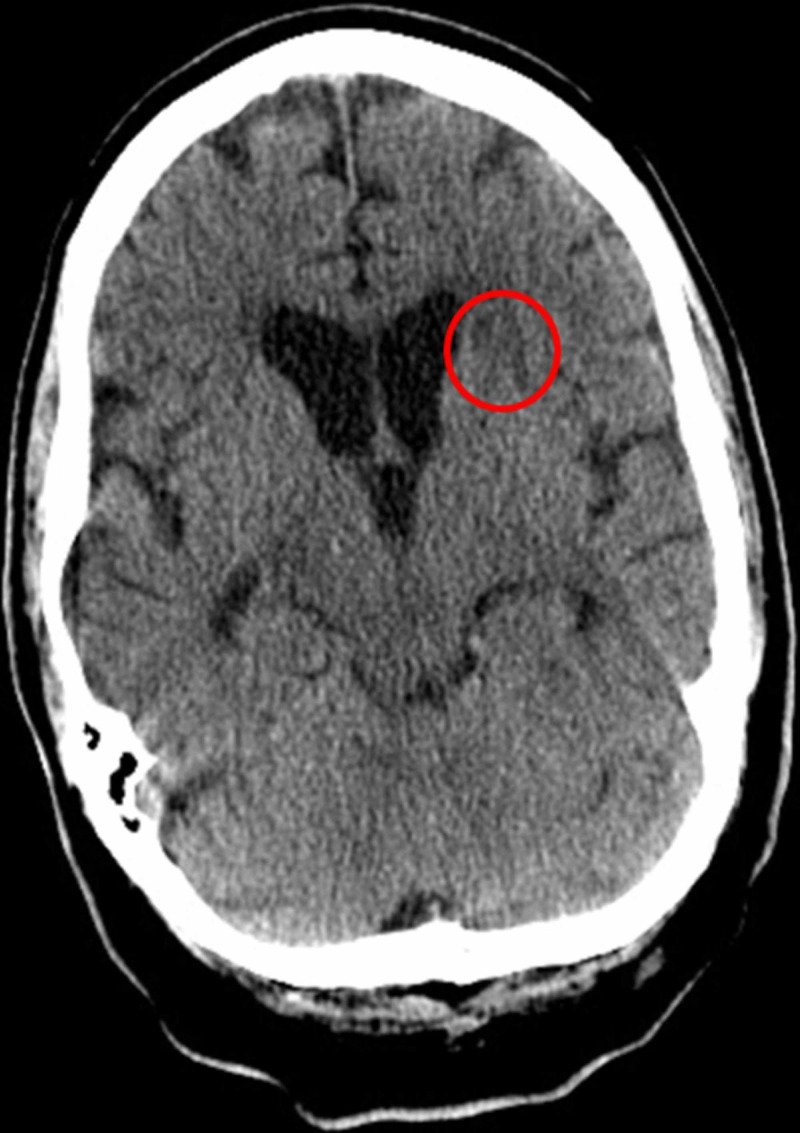
Follow-Up CT of the Brain Demonstrating Resolution of a Periventricular Abscess Follow-up axial CT of the brain shows resolution of a previously demonstrated periventricular abscess on MRI. There is a remnant of periventricular edema where the abscess was localized, shown by hypoattenuation (red circle).

A repeat CT of the brain was performed two days after pulmonary embolization, demonstrating multifocal parenchymal improvement, with complete resolution of the periventricular abscess (Figure 6).

A repeat lumbar puncture for cerebrospinal fluid analysis remained negative for infectious disease sequelae. The patient declined a follow-up MRI of the brain and was discharged nine days after intervention with home health, and six weeks of antibiotic therapy. 

At the six months follow-up, the patient reported progressive improvement since hospital discharge. He denied any systemic neurologic, gastrointestinal, or respiratory disease sequelae. At that time, the patient was counseled on continuity of care with interventional radiology and his primary care physician. He was further educated on the importance of follow-up imaging for the observation of pulmonary disease progression but unfortunately declined to pursue further assessment.

## Discussion

OWR syndrome is a rare condition with increased risk for fatal complications, due to AVMs in the hepatic, cerebral, and pulmonary vessels [3]. Regardless of rarity, interventional operators should familiarize themselves with the management options available for patients with pulmonary AVMs in the setting of OWR syndrome. The preceding case demonstrates how endovascular techniques may be utilized by physicians to prevent an acute decline in patients with systemic vascular disease. Various embolization techniques are at the disposal of an interventional radiologist; however, the literature is limited when discussing treatment for patients with pulmonary AVMs in the setting of OWR syndrome. If an operator feels inclined to intervene in this case, it is important that proper technique is used to not only successfully embolize pulmonary AVMs but to prevent severe complications [3].

Coil embolization of the feeding vessel has historically been the primary treatment of pulmonary AVMs with the anchor and scaffold technique [7]. However, this technique is limited by a significant rate of recanalization and adjacent vessel damage [7]. Therefore, interventional radiologists have recently employed novel vascular plugs with or without coils, which has resulted in more successful long-term embolization [7]. Recent studies have estimated recanalization rates when using vascular plugs to be as high as 7 percent, which is superior to a recent study listing coil recanalization rates as high as 33 percent in this setting [8]. However, it should be noted that while plugs have proven superior to coils in this case, it is not always technically possible to use larger plugs due to factors such as tortuous anatomy, small caliber size of supplying arteries, and sheath size requirement for plug deployment.

The Amplatzer plug is a nitinol-based, self-expanding mesh that is advantageous in maneuverability, repositioning, and low risk for migration [6]. One disadvantage of vascular plugs is cost; however, this is somewhat mitigated due to the requirement of fewer plugs in comparison to coils for a given AVM embolization [7]. In addition, Amplatzer vascular plugs cannot be deployed through microcatheters and necessitate the use of at least 5 French catheters for deployment, limiting the use in small-caliber vessels. The microvascular plug is a more recent development by MedtronicTM (Minneapolis, USA), which is also a nitinol-based device with a polytetrafluoroethylene membrane. This microvascular plug can be delivered by 0.027-inch or 0.038-inch microcatheters [8]. It has revealed a similar durability in comparison to Amplatzer vascular plugs, with the added benefit of improved distal device access [8]. The smaller catheter and device size have been shown to successfully treat pulmonary AVMs smaller than 2 mm and supplying arteries of 1.3 mm [8]. As expected with new devices, the cost of the microvascular plug is approximately twice that of the Amplatzer plug [8]. This device was unfortunately not available for use in this case, though maybe of beneficial use in the future.

There continues to be a risk for pulmonary AVM recanalization in the postoperative period [8]. Although the risk of recanalization is low with the modern devices described, it is important for interventional radiologists to continue following their patients for these risks [8]. This is especially important for OWR syndrome patients as there is also an elevated risk of developing new AVMs. International recommendations suggest that a post-embolization patient follow-up six to twelve months postoperatively, as well every three years thereafter, with a chest CT is suggested for observation of AMV revascularization [4]. Additionally and contrary to previous practice, all pulmonary AVMs of any size should receive intervention, as recently documented literature demonstrates that pulmonary AVMs larger or smaller than 3 mm can cause equal patient morbidity [3]. Therefore, it is suggested that interventional radiologists intervene early when capable [3].

## Conclusions

OWR syndrome is a rare condition that has been shown to carry a significant risk for AVMs, particularly in the pulmonary circulation, placing patients at risk for several life-threatening complications. Interventional embolization techniques with modern device technology have proven to be a durable and safe solution for mitigating serious disease sequelae. Interventional radiologists should remain vigilant of patients with these rare presentations in order to optimize the management of rare diseases, according to the currently suggested guidelines.
